# MiR-137 and miR-34a directly target Snail and inhibit EMT, invasion and sphere-forming ability of ovarian cancer cells

**DOI:** 10.1186/s13046-016-0415-y

**Published:** 2016-09-05

**Authors:** Peixin Dong, Ying Xiong, Hidemichi Watari, Sharon J. B. Hanley, Yosuke Konno, Kei Ihira, Takahiro Yamada, Masataka Kudo, Junming Yue, Noriaki Sakuragi

**Affiliations:** 1 Department of Women’s Health Educational System, Hokkaido University School of Medicine, Hokkaido University, N15, W7, Sapporo, 0608638 Japan; 2 Department of Gynecology, State Key Laboratory of Oncology in South China, Sun Yat-sen University Cancer Center, Guangzhou, 510060 People’s Republic of China; 3 Department of Gynecology, Hokkaido University School of Medicine, Hokkaido University, N15, W7, Sapporo, 0608638 Japan; 4 Department of Pathology and Laboratory Medicine, University of Tennessee Health Science Center, Memphis, TN 38163 USA; 5 Center for Cancer Research, University of Tennessee Health Science Center, Memphis, TN 38163 USA

**Keywords:** microRNA-137, microRNA-34a, Snail, EMT, Cancer stemness, Ovarian cancer

## Abstract

**Background:**

In ovarian cancer (OC) cells, Snail was reported to induce the epithelial-to-mesenchymal transition (EMT), which is a critical step in OC metastasis. At present little is known about controlling Snail expression in OC cells by using specific microRNAs (miRNAs).

**Methods:**

We first used a computational target prediction analysis to identify 6 candidate miRNAs that bind to the 3′-untranslated region (3′-UTR) region of the *Snail* mRNA. Among these miRNAs, two miRNAs (miR-137 and miR-34a) with a potential to regulate Snail were validated by quantitative real-time PCR, Western blot analysis, and *Snail* 3′-UTR reporter assays. We assessed the effects of miR-137 and miR-34a on EMT, invasion and sphere formation in OC cells. We also evaluated the expression of miR-137 and miR-34a in OC tissues and adjacent normal tissues and analyzed the relationship between their expression and patient survival.

**Results:**

We report that OC tissues possess significantly decreased levels of miR-137 and miR-34a and increased expression of *Snail* when compared to their adjacent normal tissues, and lower miR-137 and miR-34a expression correlates with worse patient survival. Using luciferase constructs containing the 3′-UTR region of *Snail* mRNA combined with miRNA overexpression and mutagenesis, we identified miR-137 and miR-34a as direct suppressors of *Snail* in OC cells. The introduction of miR-137 and miR-34a resulted in the suppression of Snail at both the transcript and protein levels, and effectively suppressed the EMT phenotype and sphere formation of OC cells. However, the inhibition of miR-137 and miR-34a with antisense oligonucleotides promoted EMT and OC cell invasion. Moreover, ectopic expression of Snail significantly reversed the inhibitory effects of miR-137 and miR-34a on OC cell invasion and sphere formation.

**Conclusions:**

These findings suggest that both miR-137 and miR-34a act as Snail suppressors to negatively regulate EMT, invasive and sphere-forming properties of OC cells.

## Background

The majority (75 %) of patients with ovarian cancer (OC) present with advanced disease and widely metastatic disease [1, 2]. In OC, the acquisition of invasiveness is accompanied by a shift from epithelial to mesenchymal phenotype, also called the epithelial-to-mesenchymal transition (EMT), which endows cancer cells with increased motility and invasiveness to seed metastasis and with stem cell-like properties, such as upregulation of stem cell genes (CD44 and CD133) and self-renewal ability [3, 4]. The EMT process can be initiated by a group of transcription factors including SNAIL (Snail), which repress the expression of epithelial markers (E-cadherin and ZO-1), and induce the levels of mesenchymal markers (Vimentin and N-cadherin) [5]. Therefore, identification of key actors regulating Snail expression and EMT in OC cells would have tremendous clinical utility.

Growing evidence suggests that a number of epigenetic mechanisms control the expression of genes that facilitate EMT and induce metastasis [6]. For example, microRNAs (miRNAs) have important roles in the regulation of cancer cell invasion and motility, by suppressing or promoting EMT [7]. In addition, several miRNAs [8–11] have been implicated in the regulation of Snail expression in human cancers other than OC. To date, however, little is known about controlling Snail expression in OC cells by using specific miRNAs.

In this study, we provide evidence that miR-137 and miR-34a directly bind to and down-regulate Snail levels to suppress EMT, invasion and sphere-forming ability of OC cells, and that the repression of these two miRNAs is significantly correlated with worse patient survival in OC.

## Methods

### Reagents and cell culture

Human OC cell lines (SKOV-3 and ES-2) were obtained from the American Type Culture Collection (Manassas, VA), and were cultured in DMEM/F12 medium (Invitrogen) supplemented with 10 % fetal bovine serum (FBS, Invitrogen). Normal ovarian epithelial cells (NOEC, Pricells, Wuhan, China) were cultured in Ham’s F-12 (Gibco) supplemented with 20 % FBS (Gibco). MiRNA mimic and miRNA inhibitor for miR-137 or miR-34a (30 nM, Ambion), Snail siRNA (5 nM, Ambion) and Snail cDNA plasmids (OriGene) were transfected using Lipofectamine 2000 (Invitrogen) according to the manufacturer’s protocol.

### Real-time quantitative RT-PCR (qPCR)

Total RNA was extracted using TRIzol reagents (Invitrogen) according to the manufacturer’s instructions. For mRNA and miRNA analysis, cDNAs were synthesized using the PrimeScript RT reagent kit (Takara). Then, qPCRs were performed by using the Takara SYBR Premix Ex Taq II (Takara) and the 7500 Real-Time PCR System (Applied Biosystems). The primer sequences for *Snail* [12], *E-cadherin* [12], *ZO-1* [12], *N-cadherin* [12], *Vimentin* [12], *CD44* [13], *CD133* [12] and *GAPDH* [12] have been previously reported. For miRNA analysis, qPCRs were performed using the NCode miRNA qRT-PCR analysis (Invitrogen, CA, USA). Forward primer is the exact sequence of the mature miR-137 and miR-34a. The mRNA and miRNA expression data were normalized to *GAPDH* and U6, respectively. Results were represented as the fold change relative to respective controls.

### Cell invasion assay

OC cells were grown to 50–70 % confluence and transfected as indicated. After 24 h, cells were seeded into upper chamber of Boyden chambers coated with Matrigel as described previously [14, 15]. After incubation for 24 h, the non-invading cells were gently removed with a cotton swab. Invasive cells located on the lower surface of chamber were stained with Giemsa and counted under a microscope. Relative cell invasion activities were expressed as the fold change over respective controls.

### Sphere formation assay

Single cells (1000 cells per well) were plated onto a 24-well ultra-low attachment plate (Corning) in serum-free DMEM/F12 medium supplemented with N2 plus media supplement (Invitrogen), 20 ng/ml epidermal growth factor (Invitrogen), 20 ng/ml basic fibroblast growth factor (Invitrogen) and 4 mg/ml heparin (Sigma-Aldrich). After 10 days of culture, the number of spheres larger than 50 μm was counted under an inverted microscope.

### Cell viability assay and cell apoptosis assay

Cell counting kit-8 assay (Dojindo) and Caspase-Glo 3/7 assay (Promega) were used to assess cell viability and cell apoptosis as previously reported [16]. For the cell viability assay, OC cells and NOEC cells were seeded were seeded at a density of 5 × 10^3^ per well in 96-well plates for 24 h, and then transfected with 30 nM of miR-137 or miR-34a mimic or negative control mimic (Neg mimic). After 72 h, 10 μl of Cell counting kit-8 solution was added into each well and the plates were incubated for additional 4 h at 37 °C. The UV absorbance of each sample was measured in a microplate reader at 450 nm. For the apoptotic assay, caspase-3/7 activity was analyzed in accordance with the manufacturer’s protocol. Briefly, OC and NOEC cells were seeded into 96-well plates and transfected as described above. After 72 h, an equal volume of Caspase-Glo 3/7 reagent was added into each well, and the cells were incubated at room temperature in the dark. Luminescence was measured after 3 h of incubation with the caspase substrate.

### Western blot analysis

Cells were harvested 24 h after transfections. Equal amounts of protein lysates (30 μg) were separated by 10 % SDS-PAGE for immunoblots with antibodies to Snail (Abcam), E-cadherin (GenScript), N-cadherin (BD Biosciences), Vimentin (GenScript) and GAPDH (Santa Cruz). Primary antibodies were used at a dilution of 1:1000. A horseradish peroxidase-conjugated anti-rabbit or anti-mouse immunoglobulin-G antibody was used as the secondary antibody (1:5000; Santa Cruz). Signals were detected using enhanced chemiluminescence reagents (Amersham Biosciences).

### Dual luciferase reporter assay

The *Snail* 3′-UTR luciferase vector was purchased from OriGene. Mutations in the miR-137 or miR-34a-binding sequence were generated by using the QuickChange Mutagenesis Kit (Stratagene). For luciferase assay, OC cells were seeded onto 24-well plates and transfected after 24 h with 100 ng of firefly luciferase reporter plasmid, 10 ng of Renilla report plasmid as normalization control, together with miR-137 or miR-34a mimic or Neg mimic. After 24 h, a Dual Luciferase Reporter Assay (Promega) was performed as previously reported [17]. The firefly luciferase activity was normalized to the Renilla luciferase activity.

### Clinical samples

Matched serous OC and corresponding adjacent normal ovarian tissues were obtained from 50 patients undergoing resection at the Department of Gynecology, State Key Laboratory of Oncology in South China, Sun Yat-sen University Cancer Center (Guangzhou, China). Tumor and non-cancerous tissues were confirmed histologically by Hematoxylin and Eosin staining. All samples were collected from consenting individuals according to the protocols approved by the Ethics Review Board at Sun Yat-sen University Cancer Center. All tissue samples were immediately snap-frozen in liquid nitrogen. They were kept in a -80 °C freezer and total RNA was isolated using TRIzol reagents.

### Statistical analysis

Results are expressed as mean ± s.e.m. from at least three independent experiments performed in triplicate. 2-tailed Student’s *t*-test was used for statistical analysis. The log-rank test was used for survival analysis. The value of *P* < 0.05 were considered as significant.

## Results

### MiR-137 and miR-34a are downregulated in OC tissues and OC cell lines, and decreased expression of miR-137 and miR-34a is associated with poor survival in OC patients

**Fig. 1:**
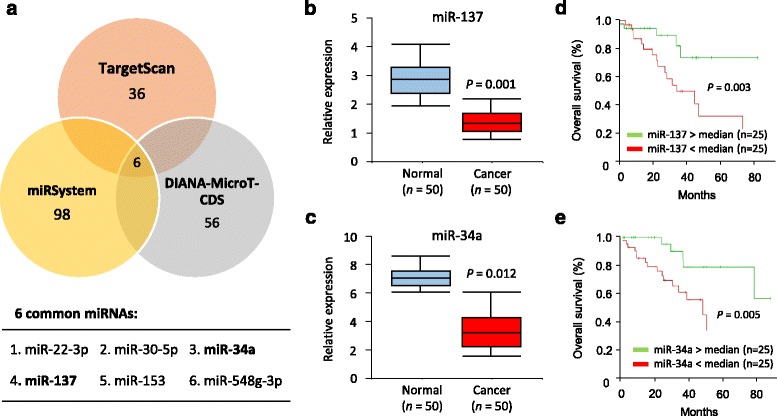
MiR-137 and miR-34a are downregulated in OC tissues and decreased expressions of miR-137 and miR-34a are associated with poor survival in OC patients. **a** Venn diagram showing the overlap of miRNAs that were predicted to bind to the *Snail* 3′-UTR by alternative algorithms (TargetScan, miRSystem and DIANA-MicroT-CDS). The 6 predicted miRNAs were common to these three algorithms. **b**, **c** qPCR analysis of miR-137 (**b**) and miR-34a (**c**) levels in 50 paired cancerous and normal tissue samples from OC patients. **d**, **e** Kaplan-Meier analysis of overall survival in 50 OC patients with high median (*n* = 25) or low median (*n* = 25) expression levels of miR-137 (**d**) or miR-34a (**e**)

To investigate miRNA regulation of Snail, we first employed multiple algorithms, including TargetScan, miRSystem and DIANA-MicroT-CDS, to screen the specific miRNAs that can target the 3′-untranslated region (3′-UTR) region of the *Snail* mRNA. These analyses revealed six common miRNAs predicted to bind the 3′-UTR of the *Snail* transcript (Fig. 1a). For example, target prediction algorithms predicted one putative binding site for miR-137 in the 3′-UTR of *Snail* (nt 444-450) and recognized one binding site for miR-34a in *Snail* 3′-UTR (nt 86–93) [10].

**Fig. 2:**
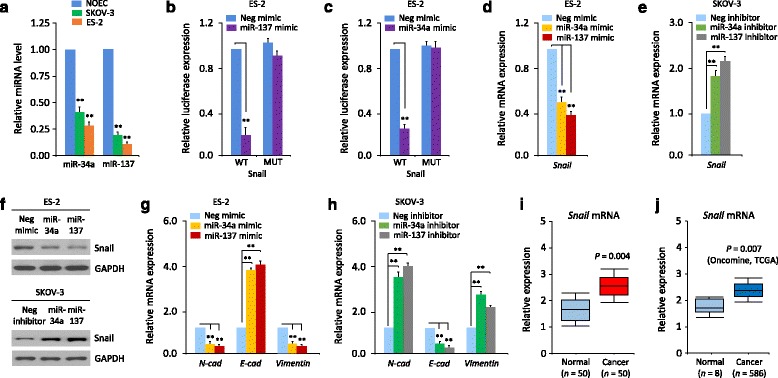
Snail is a direct target of miR-137 and miR-34a in OC cells. **a** Relative miR-34a expression in OC cell lines (SKOV-3 and ES-2) and normal ovarian epithelial NOEC cells. **b**, **c** ES-2 cells were transfected with reporter constructs containing either wild-type (WT) *Snail*, or *Snail* 3′-UTR with mutation (MUT), along with miR-137 mimic (**b**), miR-34a mimic (**c**), or negative control mimic (Neg mimic), respectively. Relative luciferase activity was measured. **d**, **e** qPCR analysis of Snail expression in OC cells after overexpression (**d**) or knockdown (**e**) of miR-137 and miR-34a. **f** Western blotting analysis of Snail expression in OC cells after overexpression or knockdown of miR-137 and miR-34a. **g**, **h** qPCR analysis of indicated mRNAs in OC cells after transient overexpression or knockdown of miR-137 (**g**) and miR-34a (**h**). **i** Relative mRNA expression of *Snail* in OC tissues and matched normal tissues. **j** Analysis of *Snail* mRNA expression using microarray (Oncomine) on normal ovary versus OC tissue. ^**^ *P* < 0.01

Next, we sought to determine whether their expression is altered in human OC by measuring the expression level of these 6 miRNAs in 50 pairs of human OC samples and adjacent normal ovarian tissues. Interestingly, the levels of all 6 miRNAs were significantly decreased in OC tissues compared with adjacent normal ovarian tissues (data not shown). Among them, miR-137 and miR-34a were the most significantly down-regulated miRNAs (Fig. 1b and c), and selected to examine their effects on Snail expression. To examine whether the downregulation of miR-137 or miR-34a has any clinical significance in OC, we analyzed the association between miR-137 or miR-34a expression and patient prognosis. OC patients population were divided into two groups based on high (*n* = 25) or low (*n* = 25) expression of miR-137 or miR-34a according to the median expression levels among OC specimens, and Kaplan–Meier curves for overall survival was plotted. Patients with lower miR-137 or miR-34a expression had significantly shorter overall survival (Fig. 1d and e). Consistent with these results, both miR-137 and miR-34a were clearly reduced in two OC cell lines (SKOV-3 and ES-2) compared with normal ovarian epithelial NOEC cells (Fig. 2a).

### Snail is a direct target of miR-137 and miR-34a in OC cells

To determine whether Snail is a direct target of miR-137 and miR-34a, we transfected a human OC ES-2 cell line (which expresses very high levels of endogenous Snail and lacks the expression of miR-137 and miR-34a) with pre-miRNA-137 or pre-miRNA-34a, together with a firefly luciferase reporter vector containing the *Snail* 3′-UTR. We found significant repression of luciferase activities by either miR-137 or miR-34a (Fig. 2b and c). In addition, these inhibitory effects of miR-137 or miR-34a on luciferase activities were eliminated following mutations targeting the predicted-binding sites of miR-137 or miR-34a within the *Snail* 3′-UTR (Fig. 2b and c), demonstrating that they bind directly to the 3′-UTR of *Snail*.

To directly assess if these miRNAs had a biological effect on Snail, mRNA and protein expression of Snail was examined in ES-2 and SKOV-3 cells following transfection of miRNA mimics or inhibitors, respectively. As expected, Snail mRNA and protein were down-regulated in ES-2 cells transfected with miR-137 or miR-34a mimic (Fig. 2d and f). In contrast, transfection with miR-137 or miR-34a inhibitor into SKOV-3 cells (which has relatively higher levels of miR-34a and miR-137) up-regulated Snail expression at mRNA and protein levels (Fig. 2e and f). Then we examined the expression of epithelial marker *E-cadherin*, mesenchymal marker *N-cadherin* and *Vimentin* in OC cells after the overexpression or knockdown of miR-137 and miR-34a, using qPCR analysis. ES-2 cells transfected with miR-137 or miR-34a mimic showed increased levels of *E-cadherin* and decreased expression of *N-cadherin* and *Vimentin* (Fig. 2g). However, transfection of SKOV-3 cells with miR-137 or miR-34a inhibitor reversed these effects (Fig. 2h).

Next, we examined the correlation between the expression of miR-137, miR-34a and *Snail* mRNA expression. We found that *Snail* mRNA level was significantly increased in OC samples compared with their non-tumor counterparts (Fig. 2i). Next, we chose to compare our data with existing published gene expression database on OC. Importantly, our analysis of the TCGA OC samples revealed significantly higher *Snail* transcript levels in OC samples, relative to normal ovaries (Fig. 2j), supporting a negative correlation between miR-137 or miR-34a and Snail expression in human OCs. Collectively, these results suggest that both miR-137 and miR-34a suppress Snail by targeting specific sites within the 3′-UTR of *Snail*.

### MiR-137 and miR-34a inhibit EMT, invasion and sphere-forming ability of OC cells

**Fig. 3:**
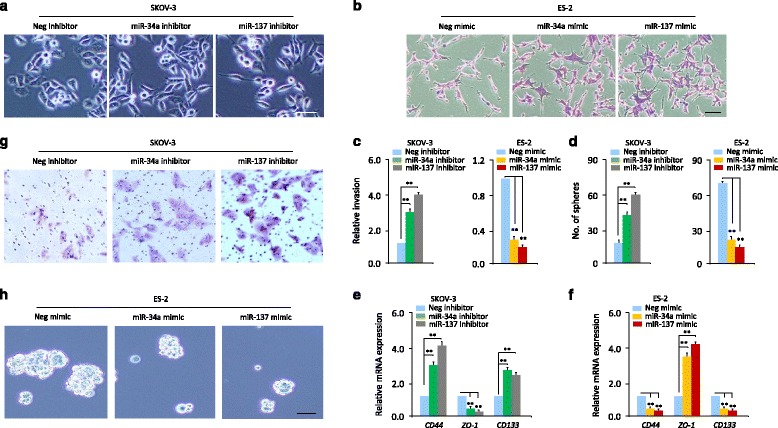
MiR-137 and miR-34a inhibit EMT, invasion and sphere-forming ability of OC cells. **a**, **b** Cellular morphology of OC cells after transient knockdown (**a**) or overexpression (**b**) of miR-137 and miR-34a. Scale bar = 200 μm (**a**) and 100 μm (**b**). **c**, **d** Cell invasion (**c**) and sphere formation (**d**) of OC cells after transient knockdown or overexpression of miR-137 and miR-34a. **e**, **f** qPCR analysis of indicated mRNAs in SKOV-3 (**e**) and ES-2 (**f**) cells after transient knockdown or overexpression of miR-137 and miR-34a. **g** Representative images of invaded SKOV-3 cells after transient transfection with miR-137 inhibitor or miR-34a inhibitor or negative control inhibitor (Neg inhibitor). **h** Representative images of spheres formed from ES-2 cells transfected with miR-137 or miR-34a mimic or Neg mimic. Scale bar =100 μm. ^**^ *P* < 0.01

We also examined the potential effects of miR-137 and miR-34a on EMT features, invasive and sphere-forming abilities of OC cells. For this, we transiently reduced or overexpressed miR-137 and miR-34a in SKOV-3 and ES-2 cells, which express relatively higher or lower expression of miR-137 and miR-34a, respectively (Fig. 2a). The down-regulation of miR-137 or miR-34a in SKOV-3 cells induced a spindle-like mesenchymal morphology (Fig. 3a), and the overexpression of miR-137 or miR-34a in ES-2 cells resulted in an epithelial morphology change (Fig. 3b). We further investigated the effects of knockdown and overexpression of these two miRNAs on EMT, OC cell invasion and sphere formation ability. Matrigel invasion assay and sphere formation assay demonstrated that knockdown of miR-137 or miR-34a using antisense oligonucleotides in SKOV-3 cells significantly promoted cell invasion and sphere formation, whereas overexpression of miR-137 or miR-34a with miRNA mimics in ES-2 cells inhibited these malignant features (Fig. 3c and d). Then we examined the expression of epithelial marker *ZO-1* and cancer stemness markers (*CD44* and *CD133*) in OC cells after the knockdown and overexpression of miR-137 and miR-34a, using qPCR assays. SKOV-3 cells transfected with miR-137 or miR-34a inhibitor showed decreased levels of *ZO-1* and increased expression of *CD44* and *CD133* (Fig. 3e). However, transfection of ES-2 cells with miR-137 or miR-34a mimic increased the mRNA expression of *ZO-1* and suppressed that of *CD44* and *CD133* (Fig. 3f). These data suggest that miR-137 and miR-34a inhibit mesenchymal characteristics and reduces self-renewal properties of OC cells.

### MiR-137 and miR-34a modulate EMT, invasion and sphere-forming ability of OC cells through targeting Snail

**Fig. 4:**
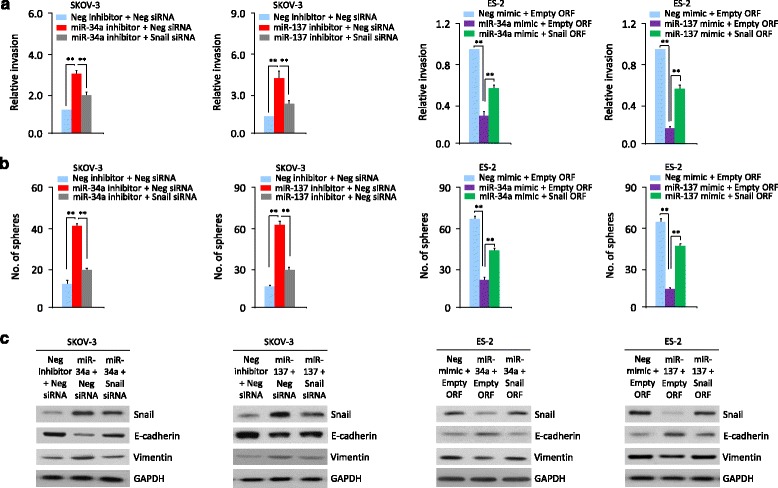
MiR-137 and miR-34a modulate EMT, invasion and sphere-forming ability of OC cells through targeting Snail. MiR-137 or miR-34a inhibitor or Neg inhibitor was co-transfected into SKOV-3 cells, together with (or without) Snail siRNA. MiR-137 or miR-34a mimic or Neg mimic was co-transfected into ES-2 cells, together with (or without) *Snail* cDNA vector lacking the 3′-UTR region. Cell invasion assay (**a**), sphere formation assay (**b**) and Western blotting analysis of indicated proteins (**c**) in OC cells treated as described above were performed. ^**^ *P* < 0.01

To further corroborate the above observations, we investigated whether silencing of Snail with specific siRNA could repress the miR-137 or miR-34a inhibitor-induced OC cell invasion and sphere formation, or whether transient over-expression of a *Snail* open reading frame (ORF) could reverse the inhibitory effects of miR-137 or miR-34a mimic on OC cell invasion and sphere formation. The miR-137 or miR-34a inhibitor-induced SKOV-3 cell invasion and sphere formation were significantly reduced by Snail siRNA (Fig. 4a and b). The overexpression of *Snail* ORF in ES-2 cells partially rescued miR-137 or miR-34a mimic-suppressed invasion and sphere formation (Fig. 4a and b). Consistent with these data, inhibiting Snail expression using siRNA in SKOV-3 cells transfected with miR-137 or miR-34a inhibitor elevated the expression of E-cadherin but reduced the expression of N-cadherin and Vimentin (Fig. 4c). Moreover, rescuing Snail expression with *Snail* ORF in the presence of miR-137 or miR-34a mimic resulted in down-regulation of E-cadherin and up-regulation of N-cadherin and Vimentin (Fig. 4c). These data suggest that miR-137 and miR-34a inhibits invasive and stem cell-like properties of OC cells by suppressing Snail expression via its 3′-UTR.

### Overexpression of miR-137 and miR-34a decreases cell viability and induces apoptosis in OC cells, without significant cytotoxicity to normal NOEC cells

**Fig. 5:**
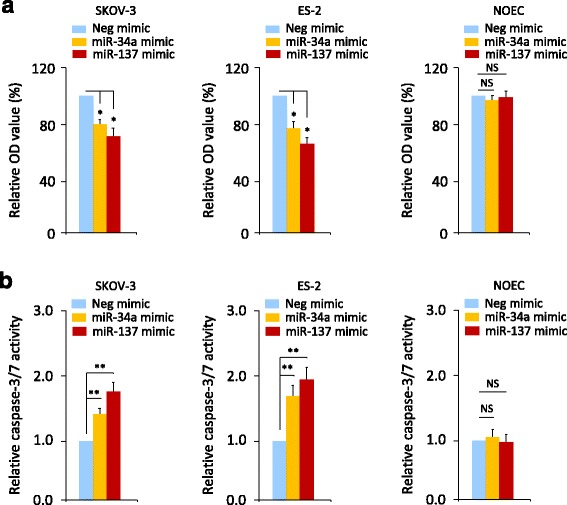
Overexpression of miR-137 and miR-34a decreases cell viability and induces apoptosis in OC cells, without significant cytotoxicity to normal NOEC cells. OC cells or normal NOEC cells were transfected with miR-137 mimic, miR-34a mimic or Neg mimic for 72 h, respectively. Cell viability assay (**a**) and cell apoptosis assay (**b**) were performed. ^*^ *P* < 0.05. ^**^ *P* < 0.01. NS: Not Significant

Next, we investigated if overexpressing miR-137 and miR-34a could affect the viability and apoptosis of OC cells and normal NOEC cells using cell viability and cell apoptosis assays. The data demonstrated that the restoration of miR-137 and miR-34a expression in ES-2 and SKOV-3 cells led to a significant reduction in cell viability and the induction of cell apoptosis, but had no significant toxicity to NOEC cells (Fig. 5), indicating the feasibility of inhibiting the growth of OC cells via the therapeutic delivery of miR-137 or miR-34a mimics, without causing significant toxicity to normal ovarian tissues.

## Discussion

Despite advances in our understanding of the mechanisms underlying Snail up-regulation, little is known about the endogenous miRNA suppressors of Snail in OC cells. We report in this study that in OC cells, both miR-137 and miR-34a act as novel tumor repressors that directly target Snail, which plays a pivotal role in controlling the various cellular functions during cancer metastasis, such as EMT, cell invasion, sphere-forming ability and chemoresistance [18–23].

We show that miR-137 and miR-34a are downregulated in OC samples, and we found a significant association between decreased miR-137 or miR-34a expression and worse patient prognosis. Furthermore, our in vitro data have confirmed that reduced expression of miR-137 and miR-34a is critical for enhanced OC cell invasiveness and self-renewal, suggesting that these two miRNAs can be potential therapeutic targets. Therefore, attenuating the oncogenic functions of Snail by the use of miR-137 and miR-34a could provide an exciting opportunity for the development of therapy against OC.

Since a single mRNA might be targeted by multiple miRNAs, we sought to newly identify crucial miRNAs that reduce the expression of Snail in OC cells. We demonstrate for the first time that miR-137 can directly repress Snail expression through its binding to the specific binding site in the 3′-UTR of the human *Snail* gene, thereby negatively regulating EMT, invasion and self-renewal of OC cells. It is interesting to note that a tumor-suppressive role for miR-137 has also been shown in a variety of human tumors [24–26]. Thus, these data and our current results reveal that, the anti-invasive effects of miR-137 described here for OC—possibly mediated by Snail suppression—might be relevant in other tumor types. Therefore, we postulate that replacing tumor suppressive miR-137 targeting Snail might be a promising approach for treating metastatic and recurrent OC.

As an important tumor suppressor, miR-34a controls the expression of a host of target proteins involved in cell proliferation, apoptosis, cancer stemness, metastasis and chemoresistance [27], and it is often down-regulated in numerous tumor types [28–32]. A previous study has shown that miR-34a acts as a suppressor of Snail in colon cancer [10], but to our knowledge, there are also opposite findings showing its tumor-promoting roles in other cancer type [33]. Whether miR-34a affects Snail expression in OC cells have remained elusive. Here, we have demonstrated that miR-34a is a direct inhibitor of Snail in OC cells. Thus, the down-regulation of miR-34a may be essential for Snail to induce EMT and OC metastasis.

Our observation that upregulating tumor suppressive miR-137 and miR-34a via miRNA mimics restored tumor suppressor activity, with successful inhibition of OC cell viability and invasiveness, provided a rationale to investigate “miRNA replacement therapy for miR-137 and miR-34a”. However, there are concerns regarding potential toxicity and off-target effects in normal tissues. We showed here that the viability and apoptosis of normal NOEC cells was not altered after transfection with miR-137 and miR-34a mimics, which display a high selectivity for killing OC cells. These findings may be explained by several mechanisms [34]. Especially, large differences in miR-137 and miR-34a levels between normal NOEC cells and OC cells might account for the tolerance of NOEC cells to these two miRNAs. The in vivo anti-tumor efficacy and toxicity of miR-137 and miR-34a warrants further investigation.

## Conclusions

We identified a new mechanism whereby decreased expression of both miR-137 and miR-34a contributes to enhanced Snail levels, which in turn promotes EMT, invasion and sphere-forming of OC cells. Our results suggest that these two miRNAs might become candidate targets for the treatment of Snail-overexpressing OC.

## Abbreviations

EMT: Epithelial-to-mesenchymal transition
miRNAs: microRNAs
